# Recovery patterns and physics of the network

**DOI:** 10.1371/journal.pone.0245396

**Published:** 2021-01-19

**Authors:** Alireza Ermagun, Nazanin Tajik

**Affiliations:** 1 Department of Civil and Environmental Engineering, Mississippi State University, Mississippi State, MS, United States of America; 2 Department of Industrial and Systems Engineering, Mississippi State University, Mississippi State, MS, United States of America; University of Zilina, SLOVAKIA

## Abstract

In a progressively interconnected world, the loss of system resilience has consequences for human health, the economy, and the environment. Research has exploited the science of networks to explain the resilience of complex systems against random attacks, malicious attacks, and the localized attacks induced by natural disasters or mass attacks. Little is known about the elucidation of system recovery by the network topology. This study adds to the knowledge of network resilience by examining the nexus of recoverability and network topology. We establish a new paradigm for identifying the recovery behavior of networks and introduce the recoverability measure. Results indicate that the recovery response behavior and the recoverability measure are the function of both size and topology of networks. In small sized networks, the return to recovery exhibits homogeneous recovery behavior over topology, while the return shape is dispersed with an increase in the size of network. A network becomes more recoverable as connectivity measures of the network increase, and less recoverable as accessibility measures of network increase. Overall, the results not only offer guidance on designing recoverable networks, but also depict the recovery nature of networks deliberately following a disruption. Our recovery behavior and recoverability measure has been tested on 16 distinct network topologies. The relevant recovery behavior can be generalized based on our definition for any network topology recovering deliberately.

## Introduction

Networks have become a ubiquitous tool in understanding both natural and engineered systems, including, climate systems [1], physiological systems [2], civil infrastructure systems [3, 4], social interactions [5–8], and biochemical reactions [9]. As a representation of the interaction patterns in complex systems, networks equip scientists to manifest how systems exhibit similarities in the organization of their structure [10–13], in their complex features [14], and in their resilience [14–16]. In a progressively interconnected world, the loss of system resilience has consequences for human health [17], the economy [18], and the environment [19], yielding in the assessment of the system’s dynamics and topology from meso- and macro-scale perspectives. The failure of an individual component may or may not influence the performance of a system, depending on the topology and the intensity of the interactions among the network components. A scale-free topology, for example, is robust against random attacks, but is vulnerable to localized attacks targeted at hubs [20].

A large proportion of research has exploited the science of networks to explain the resilience of complex systems against random attacks [12, 21, 22], malicious attacks [23–25], and localized attacks induced by natural disasters or mass attacks [26–28]. While there is no singular definition of network resilience, it is frequently interpreted as the ability of a network to prepare, respond, and recover from heterogeneous and dynamic sets of hazards [29]. A network, when affected by an attack, experiences three states: (1) original state, (2) disrupted state, and (3) recovered state. Transitioning between the original state and the disrupted state forms the system disruption, and transitioning between the disrupted state and the recovered state constitutes the system recovery. The mechanism of recovery, in nature, happens spontaneously or deliberately [30]. In fact, a damaged component of the network returns to a normal condition either by itself or through deliberate action. Previous research has contributed to the interpretation of system disruption by percolation theory [11, 31, 32] and critical component identification [33, 34]. Theory aside, the network topology has long been implicated in dictating the properties of network resilience [20, 35, 36]. Little is known about the elucidation of system recovery by the physics of the network. How does a system transition from the disrupted state to the recovered state? What characteristics affect the degree of convexity in recoverability trajectories? What are the topological characteristics that facilitate or impede the transition?

Here, we establish a new paradigm for measuring the recovery of networks and identifying the interdependence between physics of the network and recoverability. Our intention, as the overarching goal of the current study, is to equip scientists and practitioners with the knowledge and tools necessary to characterize the nexus of network topology and recoverability. This is achieved through an integrated study of networks with different topological patterns and sizes in the face of attacks, which drastically compromise the performance of networks by demolishing a set of links or the total network. This knowledge is needed to determine better techniques to avoid cascading failures and improve the dissemination of demand through the network [37, 38]. The research objectives to help achieve our overarching goal are (1) gain a fundamental understanding of the recovery response of networks with different size and topology, (2) quantify the relationship between recoverability and network topology characteristics, and (3) determine how the evolving behavior of networks influences the recovery response and recoverability of networks.

We develop and test our algorithm on 16 directed and unweighted networks with distinct topologies in ten different sizes. Previous research exercised random recovery, greedy recovery, and local optimal strategies to recover disrupted networks [39, 40]. Unlike the random recovery that restores damaged links randomly, greedy recovery and local optimal strategies repair the network taking into consideration the network functionality. The link restoration selection in the greedy recovery strategy assures maximizing the network functionality in each step, which brings high computational complexity. Local optimal strategies reduce the computational expense by weighting each link. Our proposed model uses a combination of branch-and-bound and dual simplex methods to find the optimal sequence of links to recover. Despite heuristic strategies, the proposed exact methods guarantee the maximum elevation in the functionality of the evolving network topology over the recovery horizon as the simplex method sifts through all feasible solutions one at a time to find the global optimal solution. The computational expenses, however, increase exponentially as the network size increases. The algorithm practices the optimal restoration plan for a completely disrupted network by forming the best sequence of disrupted links. The functionality of the algorithm is calibrated to recover a sequence of links, each reconnecting the maximum possible origin-destination (OD) pairs.

**Fig 1:**
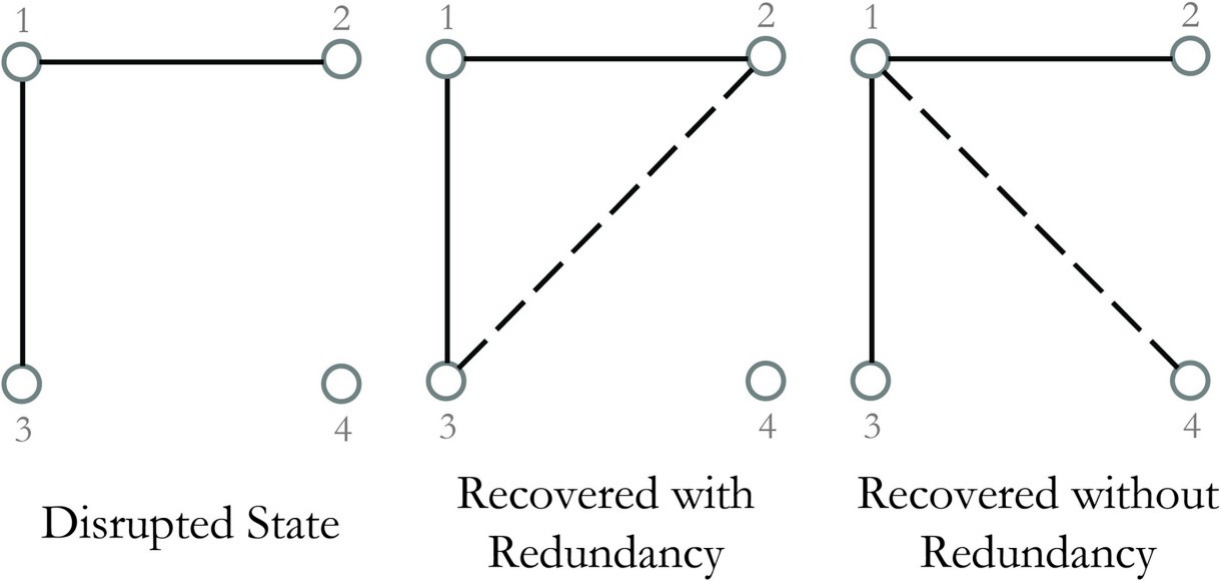
Schematic of two recovery options in a toy network.

The choice of which link to recover in each step affects the recoverability trajectory of the residual network, as restoration might augment the system performance or increase the redundancy of already recovered paths. This is illustrated in Fig 1. This figure exemplifies the two recovery alternatives on a complete graph with four nodes and two recovered links. If link (2,3) is recovered, the system experiences redundancy rather than performance enhancement. If link (1,4), (3,4), or (2,4) is recovered, however, the system is fully connected and delivers the total initial demand. Although an exhaustive search is the most guaranteed method to recover without redundancy, it is not a timely solution for large networks. The complexity of the search proliferates by *O*(*n*!) with an increase in the number of links, forming NP-hard problems. The reader is referred to S1 File for the mathematical representation of the induction proof. Exact methods cannot solve NP-hard problems for large scale instances. For small to medium scale instances, however, the Gurobi solver used in this study obtains the global optimal solution by employing concurrent simplex and barrier algorithms.

Our empirical findings demonstrate how and to what extent adding redundant paths elevates the robustness of the network. In turn, this adds to the knowledge of identifying the performance of residual network topologies following the recovery of each component. The remainder of this study is organized as follows. First, the recoverability measure is introduced. Second, a recoverability optimization algorithm is proposed along with the charactersitics of the networks used for the analysis. Third, the analytical and numerical results are discussed in three separate parts to understand (1) how the trajectory of recovery is a function of topology and size, (2) the relationship between the recoverability measure and the network topology and size, and (3) how the recoverability measure is identified by connectivity and accessibility measures. This is followed by a discussion and conclusion.

### An introduction to recoverability measure

A network is a mathematical representation of a real-world system. In graph theory, an unweighted directed network is defined as an ordered pair *G* = (*N*,*L*), where *N* is the set of nodes and *L* is the set of links. We define $\varphi_{\left|L\right|}^{G}$ as the performance of network *G* = (*N*,*L*) corresponding to the original state, where |*L*| number of links are operational in the network and the total initial demand is delivered between *OD* pairs. Following the elimination of |*J*| links, where *J*⊆*L*, the performance of network *G* = (*N*,*L*) diminishes to $\varphi_{\left|L|-|J\right|}^{G}$ and the network transitions from the original state to the disrupted state. A recovery process, if it happens, restores the network from a disrupted state to a recovered state by increasing the performance of the network from $\varphi_{\left|L|-|J\right|}^{G}$ to $\varphi_{\left|L|-|J|+|I\right|}^{G}$. The network is fully recovered if $\varphi_{\left|L|-|J|+|I\right|}^{G}$ equals $\varphi_{\left|L\right|}^{G}$. Here, $\varphi_{\left|L|-|J|+|I\right|}^{G}$ is the performance of the recovered state with the recovery of |*I*| links, where *I* is the set of recovered links and *I*⊆*J*. Mathematically, we examine the recovery of network *G* following the restoration of *i*^*th*^ link, where *i*∈*I*, by measuring the total delivered demand after the recovery of *i*^*th*^ link over the total initial demand in the network.

$$R_{G}(i)=\frac{\varphi_{|L|-|J|+i}^{G}}{\varphi_{\left|L\right|}^{G}}$$ (1)

**Fig 2:**
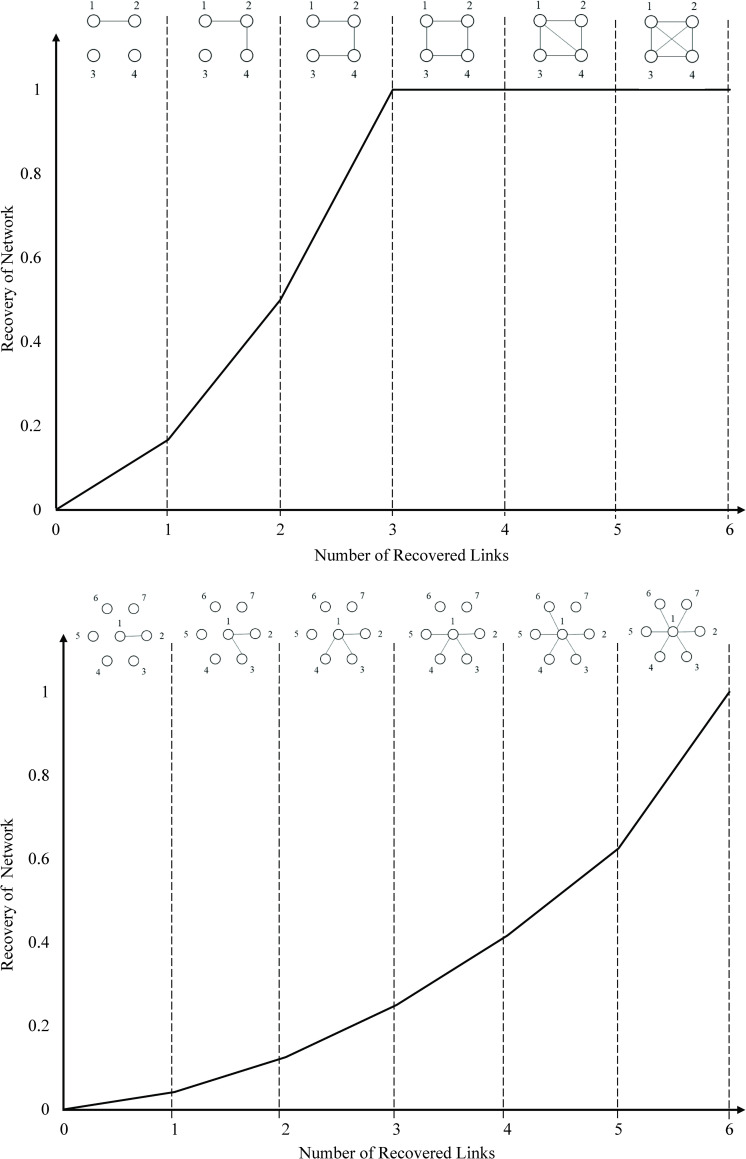
Recovery trajectory of toy complete and hub-and-spoke topologies. This figure portrays the trajectory of the recovery process for two toy networks with different topologies: (A) Complete topology and (B) Hub-and-Spoke topology.

This ratio indicates (1) the system is fully recovered if the demand delivered through the network equals the total initial demand, and (2) no recovery without redundancy is exhibited if there is no change in the delivery of demand following the disruption. As depicted in Fig 2, the topology of the recovered state in which the performance of the network equals the original state performance is not necessarily the same as the original state, and might be similar or different from the topology of the original state. Looking at complete network topology, it is inferred that the performance of the network escalates from $\varphi_{\left|L|-|J\right|}^{G}$ to $\varphi_{\left|L\right|}^{G}$ by restoring the third disrupted link. Related but not alike, the hub-and-spoke topology requires the restoration of all links to reach $\varphi_{\left|L\right|}^{G}$.

Here, we introduce $R_{G}(N,L)$ as the recoverability measure of a network with |*N*| nodes and |*L*| links, which manifests itself through the number of links required to reach the original state performance following a disruption. Mathematically, the recoverability measure is defined in Eq 2. In this equation, |*S*| is the cardinality of sets of required links to reach the original state performance following a disruption and |*L*| is the cardinality of sets of links at the original state of the network. As alluded to in Fig 1, |*S*| is not necessarily the same as |*L*|, and the ratio is a function of topology.

$$R_{G}(N,L)=1-\frac{\left|S\right|}{\left|L\right|},S\subseteq L$$ (2)

The following considerations are important. First, the recoverability measure indicates the proportion of links that can remain disrupted while the network delivers the total initial demand. Second, the minimum value of $R_{G}(N,L)$ equals zero, obtained when $\frac{\left|S\right|}{\left|L\right|}=1$. This happens when the network is fully disrupted and requires the restoration of all links to reach $\varphi_{\left|L\right|}^{G}$. Third, the maximum value of $R_{G}(N,L)$ equals one, obtained when |*S*| = 0. This happens when the network requires the restoration of no links to reach $\varphi_{\left|L\right|}^{G}$.

## Materials and methods

Following a disruptive event, a set of links, *L*′⊆*L*, are adversely impacted, deteriorating the performance of the network to $\varphi_{\left|L|-|L^{\prime }\right|}^{G}$. Here, we introduce an optimization model that provides a sequential link recovery plan to maximize the total delivered demand after the recovery of each disrupted link. The objective function is concise:

$$max\sum_{l=1}^{\left|L^{\prime }\right|}R_{G}(l)$$ (3)

To distribute the demand in network *G*, the model represents a set of flow balance equations. For each pair of origin-destination, Eq 4 distributes one unit of demand from the origin node to the destination node. Eq 5 assures the distributed demand reaches to its corresponding destination, while Eq 6 assures that no transshipment node absorbs the demand or adds any additional unit of demand to the origin-destination path.

$$\sum_{k:(i,k)\in A}f_{ijikl}-\sum_{k:(k,i)\in A}f_{ijkil}\leq 1\;\forall (i,j)\in OD,\;l=1,\ldots ,|L|$$ (4)

$$\sum_{j:(k,j)\in A}f_{ijkjl}-\sum_{j:(j,k)\in A}f_{ijjkl}=\omega_{ijl}\;\forall (i,j)\in OD,\;l=1,\ldots ,|L|$$ (5)

$$\sum_{j:(k,j)\in A}f_{ijkjl}-\sum_{j:(j,k)\in A}f_{ijjkl}=0\;\forall (i,j)\in OD,\;l=1,\ldots ,|L|,k\in N-\{i,j\}$$ (6)

The model defines *f*_*ijhkl*_, a continuous variable, as the flow of link (*h*,*k*)∈*L*, distributing the origin-destination path (*i*,*j*)∈*OD*, and *ω*_*ijl*_, a continuous variable, as the flow reaching destination node *i*∈*N* from origin node *j*∈*N* after the recovery of *l*^*th*^ link. $P_{2}^{\left|N\right|}$ represents the capacity of each link and is sufficient enough to carry total demand. To complete the set, the model assures the flow does not exceed the capacity of links and demand node and formulates it as expressed in Eq 7 and Eq 8. The network performance after the recovery of *l*^*th*^ link is calculated as the total delivered demand in the residual network after the *l*^*th*^ link.

$$0\leq \omega_{ijl}\leq 1\;\forall (i,j)\in OD,\;l=1,\ldots ,|L|$$ (7)

$$0\leq f_{ijhkl}\leq P_{2}^{\left|N\right|}\;\forall (i,j)\in OD,\forall (l,k)\in A,l=1,\ldots ,|L|$$ (8)

$$\varphi_{|L|-|L^{\prime }|+l}=\sum_{(i,j)\in OD}\omega_{ijl}\;l=1,\ldots ,|L|$$ (9)

In the recovery schedule problem, *α*_*hkl*_, a binary variable, sets disrupted link (*h*,*k*)∈*L*′ as the *l*^*th*^ recovered link in network *G*. Aligned with the value of *α*, the recovered links return to the system in sequential order, using:

$$\sum_{(i,j)\in SD}f_{ijhkl}\leq P_{2}^{\left|N\right|}\sum_{s=1}^{l}\alpha_{hkl}\;\forall (i,j)\in OD,\forall (l,k)\in L^{\prime },l=1,\ldots ,|L^{\prime }|$$ (10)

$$\sum_{l=1}^{\left|L^{\prime }\right|}\alpha_{hkl}=1\;\forall (l,k)\in L\prime$$ (11)

$$\sum_{(h,k)\in A^{\prime }}\alpha_{hkl}=1\;l=1,\ldots ,|A^{\prime }|$$ (12)

In general, the recovery model scales as *O*(|*L*′|^2^), where |*L*′|^2^ is the number of binary variables used in the computational domain. The quadratic running time increases the CPU time drastically as the number of nodes, and consequently, links increases. To reduce the computational time, we relax the binary decision constraint and provide an initial feasible solution for the model. The initial solution, along with the model results in an optimal recoverability trajectory in a timely manner.

**Fig 3:**
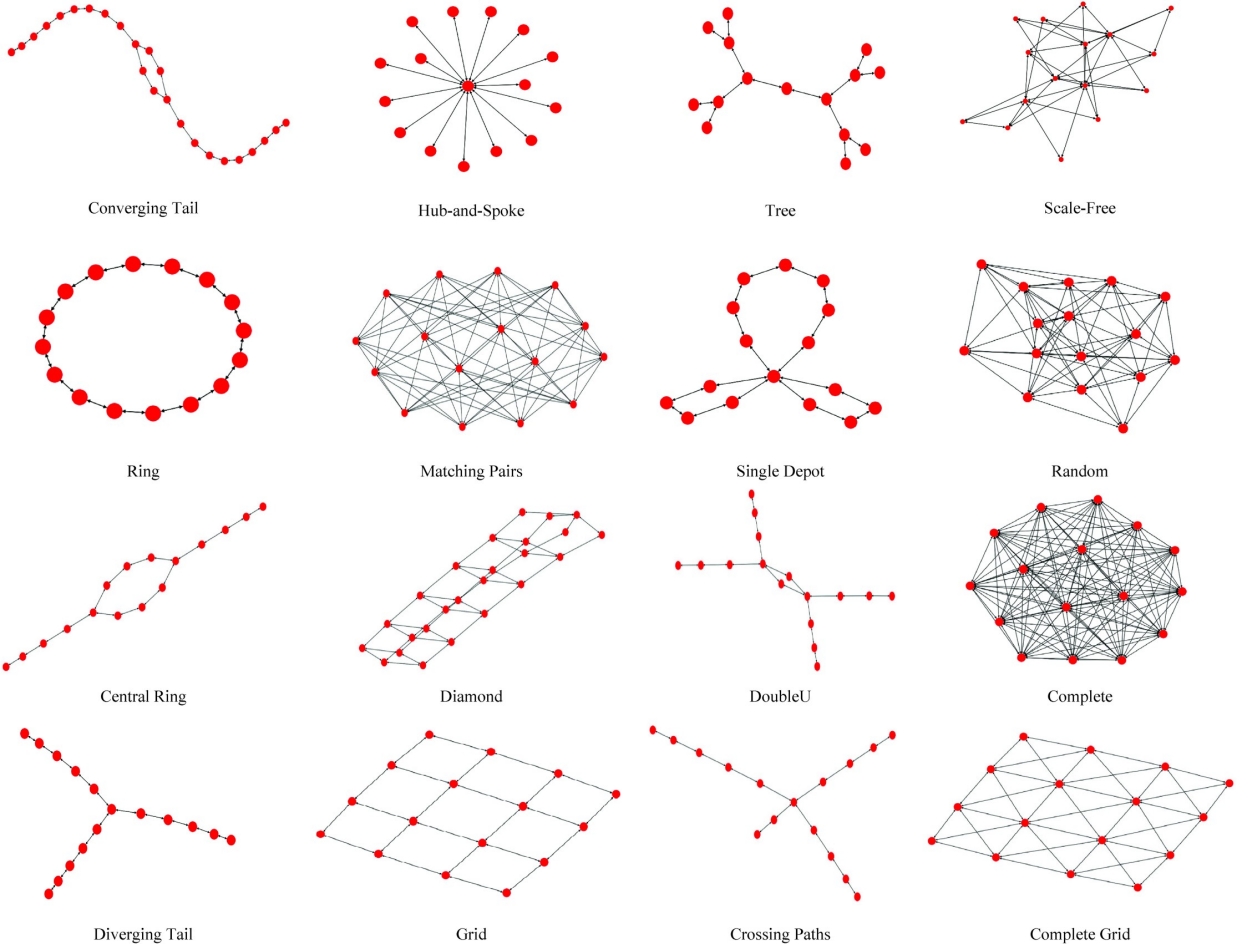
Network topology and extrapolation with 16 nodes used in the current research.

Despite the binary constraint relaxation, the solution time of the linear model increases polynomially as the number of disrupted links increases. For example, for a totally disrupted complete network with 30 nodes, there are 870 direct links to be recovered. The problem then has the solution time complexity of *O*(870*log*^870^) on average. Without loss of generality, we limit the recovery model to be tested on 16 network topologies with 8, 10, 12, 14, 16, 18, 20, 22, 24, and 26 nodes. Fig 3 depicts selected network topologies with 16 nodes as a schematic example. Alongside the number of nodes, a set of properties are required to generate the structure of each network topology. For example, we use Barabási–Albert model [41] to generate a scale-free network. The model needs to have the number of required links to attach a new node to existing nodes as well as the total number of nodes. Also, to generate random networks, we need to identify the probability of link creation alongside the number of nodes. The production procedure starts with positioning nodes on a plane and then connecting each node to the growing network iteratively following the network properties. Table 1 represents the property of each network topology for each network size.

**Table 1:** Number of links and nodes for different network topologies.

Network Topology	Network Size									
	|N| = 8	|N| = 10	|N| = 12	|N| = 14	|N| = 16	|N| = 18	|N| = 20	|N| = 22	|N| = 24	|N| = 26
Divergin Tail	7	9	11	13	15	17	19	21	23	25
DoubleU	8	10	12	14	16	18	20	22	24	26
Hub-and-Spoke	7	9	11	13	15	17	19	21	23	25
Tree	7	12	12	14	14	20	20	22	24	26
Scale Free	15	21	27	33	39	45	51	57	63	69
Random	14	22	30	52	47	66	77	146	115	131
Single Depot	10	12	14	16	18	20	22	24	26	28
Crossing Path	7	9	11	13	15	17	19	21	23	25
Matching Pair	16	25	36	49	64	81	100	121	144	169
Ring	8	10	12	14	16	18	20	22	24	26
Central Ring	8	10	12	14	16	16	20	22	24	26
Complete	28	45	66	91	120	153	190	231	276	325
Converging Tail	8	9	12	14	16	18	20	22	24	26
Diamond	12	12	20	20	28	28	36	36	44	44
Grid	10	13	17	19	24	27	31	31	38	37
Complete Grid	20	21	29	31	42	41	55	51	68	61

Note: Each cell represents number of links.

For each network, Algorithm 1 captures the set of nodes, the set of links, a list of origin-destination paths, number of cycles, the average degree of each node, and the betweenness centrality of each link. The algorithm then uses this information to obtain diameter, average Shimbel, beta, and gamma indices. The definition of topological indices is summarized in Table 2.

**Table 2:** Definition of network topology measures used in the current research.

Index	Formula	Definition
*Connectivity*		
Beta	$\beta =\frac{\left|L\right|}{\left|N\right|}$	Ratio of number of links to number of nodes.
Gamma	$\gamma =\frac{\left|L\right|}{\left|N|(|N|-1\right)}$	Ratio of number of links to maximum possible number of links in a directed network.
*Accessibility*		
Diameter	*D* = *max*(*d*_*ij*_)	The maximum distance of all shortest paths between all origin-destination pairs in the network.
Average Shimbel	$A_{}=\frac{\sum_{i=1}^{\left|N\right|}\sum_{j=1,i\neq j}^{\left|N\right|}d_{ij}}{\left|N\right|}$	The average of the length of shortest paths connecting each node to the others.
Betweenness Centrality	$BC_{ij}=\frac{\sigma_{hk}(i,j)}{\sigma_{hk}}$	Number of times a node is crossed by shortest paths in a network. *σ*_*hk*_ is the total number of paths between nodes *h* and *k*. *σ*_*hk*_(*i*,*j*) is the number of paths between nodes *h* and *k* that include link (*i*,*j*).

In the next step, the algorithm disrupts all links and feeds the residual network to the recoverability model. The proposed model represents the optimal recoverability trajectory of each network, the minimum number of links required to reach its full performance, and the area under the recoverability curve.

Algorithm 1. Recoverability Trajectory model

1: Input

    *U* = {8,10,12,14,16,18,20,22,24,26}

    Paths={},Sub_Paths={},G={},B={}

2: for *u* in U do

    ***Networks with u nodes and u−1 links*:**

    $G\leftarrow G_{Tree}(u,1)\to$ Tree network with one main branch

    $G\leftarrow G_{SingleDepot}(u,3)\to$ Traveling Salesman routing network with three main paths

    $G\leftarrow G_{Ring}(u)\to$ Ring network

    $G\leftarrow G_{ConvergingTail}(n,u)\to$ Converging Tail networks with a Ring with size *n* and two branches with *a*, and b nodes, where *a*+*b* = *u*−*n* and *n*<u

    $G\leftarrow G_{CrossingPath}(n,u)\to$ Crossing Path network with two paths with *n*, and *u*−*n* nodes, where u<*n*

    $G\leftarrow G_{CenralRing}(n,u)\to$ Converging Tail networks with a Ring with size *n* and four branches with *a*,*b*,*c*, and *d* nodes, where *a*+*b*+*c*+*d* =*u*−*n*, and *u*<*n*

    $G\leftarrow G_{DoubleU}(u)\to$ DoubleU network with a 4 nodes ring and two bi branches with *a*,*b*,*c*, and *d* nodes, where *a*+*b*+*c*+*d* = *u*−4

    $G\leftarrow G_{DivergingTail}(n,m,u-n-m)\to$ Diverging Tail with three branches with *n*,*m*, and *n*−*m* nodes, where *n*+*m*<*u*

    ***Networks with u nodes and l links (l≠u*−1):**

    $G\leftarrow G_{Grid}(n,m)\to n\times m$ grid networks with *n*×*m* nodes and 2*mn*−*m*−*n* links, where *n*×*m* = u

    $G\leftarrow G_{ScaleFree}(u,\varsigma ,\vartheta ,\xi )\to$ Scale-Free network with *i* nodes, where *ς*+*ϑ*+*ξ* = 1

    $G\leftarrow G_{CompleteGrid}(n,m)\to n\times m$ complete grid network, where *n*×*m* = *u*

    $G\leftarrow G_{Diamond}(m^{*})\to$ Diamond network with m* nodes, where $m^{*}=Argmin_{m>0,4m<n}\left(Remainder(\frac{n}{4m})\right)$

    $G\leftarrow G_{Random}(u,p)\to$ Random network with the node connection probability *p*

    $G\leftarrow G_{MatchingPairs}(n,m)\to$ Matching Pair network with *n* and *m* nodes lined up parallelly, and *n*×*m* links

    $G\leftarrow G_{Complete}(u)\to$ Complete network with *u* nodes and $\frac{u\times (u-1)}{2}$ links

    $G\leftarrow G_{Hub.Spoke}(u+1)\to$ Complete network with *u*+1 nodes and *u* links

3:    for G∈G do

4:        *N*←*G*.*nodes*()→ Set of nodes

5:        *L*←*G*.*edges*()→ Set of links

6:        *Connectivity*←***DFS***(*G*)→ Depth First Search Algorithm (*DFS*) finds total number of cycles in network *G*

7:        $Average.Degree\leftarrow \frac{\left|L\right|}{u}\to$ Ratio of twice the number of links to number of nodes in network *G*

8:        $\beta_{}\leftarrow \frac{\left|L\right|}{u}\to$ Ratio of number of links to number of nodes

9:        $\gamma_{}\leftarrow \frac{\left|L\right|}{u(u-1)}\to$ Ratio of number of links to the maximum possible number of links in network *G*

10:        for *k* = 1,…,*h* do

11:            *Sub_Paths* = {}

12:            for *κ* = 1,…,*h* do

13:                *Sub*_*Paths*[*κ*]_ = *Dijkstra*(*k*,*κ*)→ Dijkstra Algorithm finds all origin-destination paths, *d*_*ij*_,(*i*,*j*)∈*L* in network *G*

14:            *Paths*[*k*] = *Sub_Path*

15:        for *i* = 1,…,|*N*| do

16:            for *j* = 1,…,|*N*| do

17:                *count* = 0

18:                    for *k* in Paths do

19:                        if (*i*,*j*) in *k* do

20:                        *count* = *count*+1

21:                    *B*[*i*,*j*]←*count*

22:        $Betweenness\;Centrality\leftarrow \frac{\sum_{(i,j)\in L}B[i,j]}{\left|L\right|}$

23:        $Diameter\leftarrow \frac{d_{ij}}{P_{2}^{\left|N\right|}}$

24:        $Average\;Shimbel\leftarrow \frac{\sum d_{ij}}{(u-1)u}$

25:        *φ*_*l*_←*Recovery formulation* (*G*,*L*′ = *L*)

26:        $l^{*}\leftarrow \underset{=1,\ldots ,L^{\prime }}{\min}\left(l\right)\to$ the minimum number of links required to be recovered to result in, *R*(l*) = 1

## Analytical and numerical results

### Return to recovery

**Fig 4:**
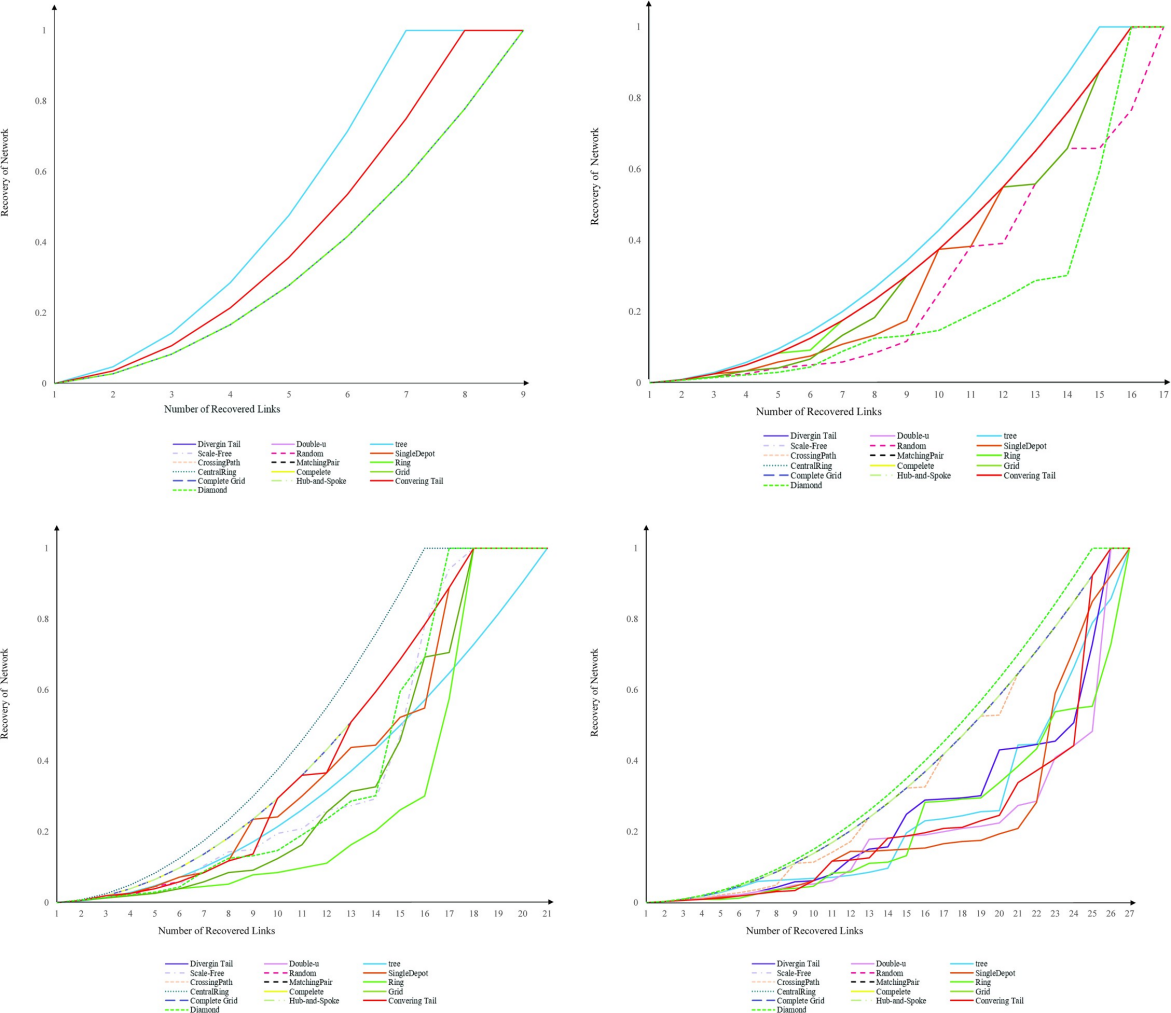
Recovery trajectory of 16 network topologies. (A) 8 nodes, (B) 14 nodes, (C) 20 nodes, and (D) 26 nodes.

We pinpoint how the trajectory of recovery is a function of topology and size, and encapsulate how the topology either impedes or facilitates the recovery of the network by studying 16 network topologies. The topologies selected in this study exist in climate systems, physiological systems, civil infrastructure systems, social interactions, and biochemical reactions. We investigate the trajectory of the recovery for each network topology from complete shutdown, where all links are disrupted, to full operation, where no demand remains unsatisfied. For modeling, we choose a range of network sizes starting from 8 nodes to 26 nodes. Regardless of the size and topology of a network, we assign one unit of demand to each origin-destination pair. The cost of each link equals one. Our algorithm recovers the disrupted network in a sequential order by maximizing the residual network performance following the recovery of each disrupted link. Fig 4 depicts the trajectory of recovery for 16 network topologies with four sizes of 8, 14, 20, and 26 nodes. The reader is referred to S1 Fig for all sizes. Two observations are noted. First, the return to recovery happens with an increasing return rate, while the return shape depends on the topology and size of the network. This confirms that as recovery proceeds, the next recovered link brings the shortest paths back to the system. In small sized networks with 8 nodes, the return to recovery exhibits homogeneous recovery behavior over topology and our 16 topologies merge into three clusters. The first cluster includes converging tail, diverging tail, hub-and-spoke, complete, central ring, ring, matching pairs, crossing path, single depot, random, scale-free, and double-u topologies. The second cluster includes diamond, grid, and complete grid topologies. The third cluster includes only tree topology. The return shape is dispersed with an increase in the size of the network. For some topologies, the increase in the size of a network disturbs the uniformity of recovery rate function, starting with the slow increase in the rate of recovery, and halfway through the recovery process, proceeding with a steep recoverability trajectory. Concretely speaking, the increase in the number of nodes, for some topologies, means the increase in the average length of the shortest paths, and consequently in the number of required links to recover them. This return to recovery is observed in converging tail, ring, central-ring, and single-depot networks.

### Recoverability and network topology

**Fig 5:**
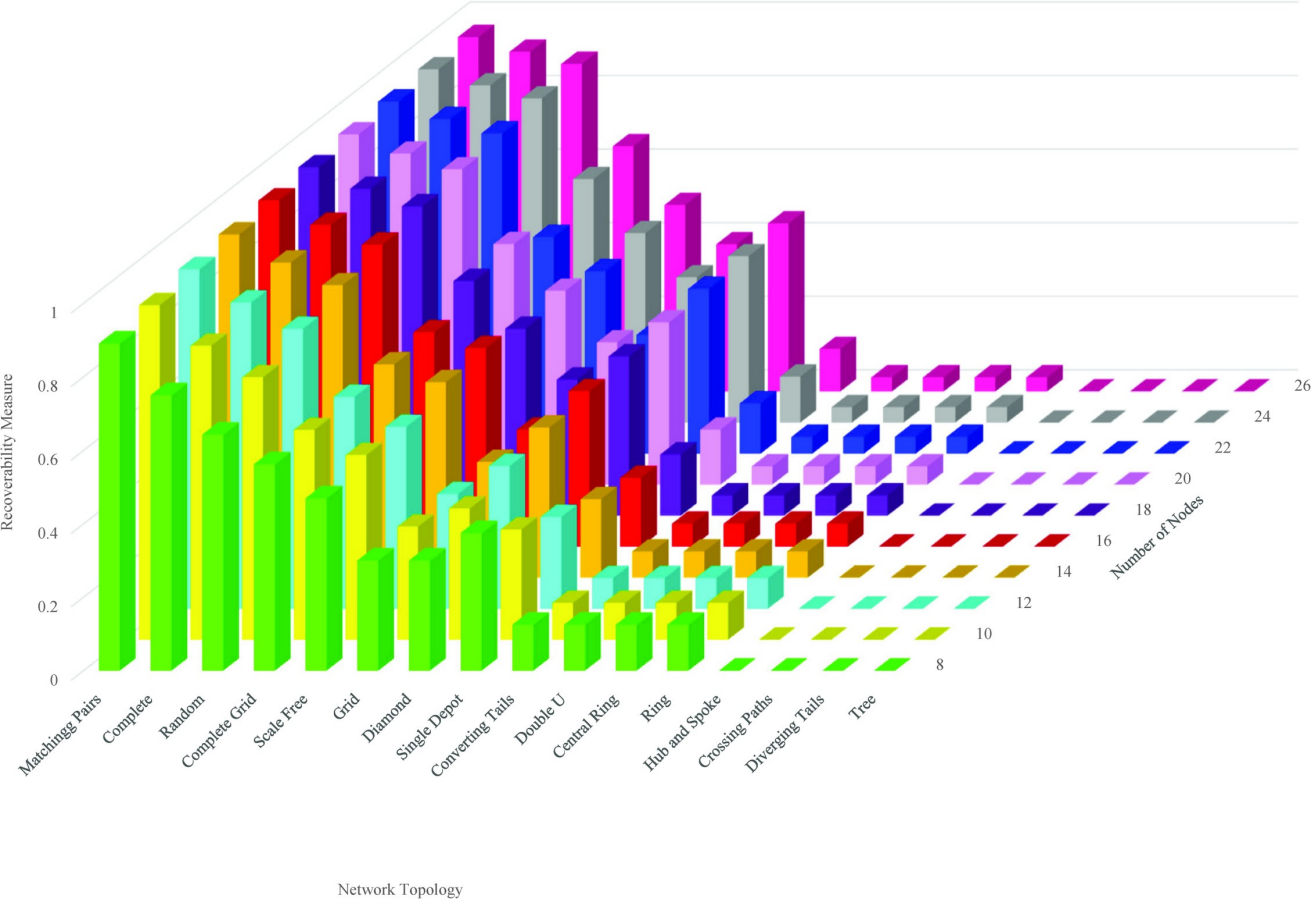
Recoverability measure over multiple size and topology.

We elucidate the relationship between the recoverability measure and the network topology and size. Unlike the trajectory of recovery, the recoverability measure, regardless of size, exhibits almost homogeneous recovery behavior over topology. This is observed in Fig 5. This figure represents the variation in the recoverability measure over multiple sizes and topologies of the network. The recoverability measure is the highest for matching pairs topology. This is followed by complete and random topologies. The lowest recoverability measure goes to tree, diverging tails, crossing paths, and hub-and-spoke, showing no superior recovery performance over size. The recoverability measure of these networks is equal to 0. It means 100% of the network should be recovered to deliver the total initial demand.

**Fig 6:**
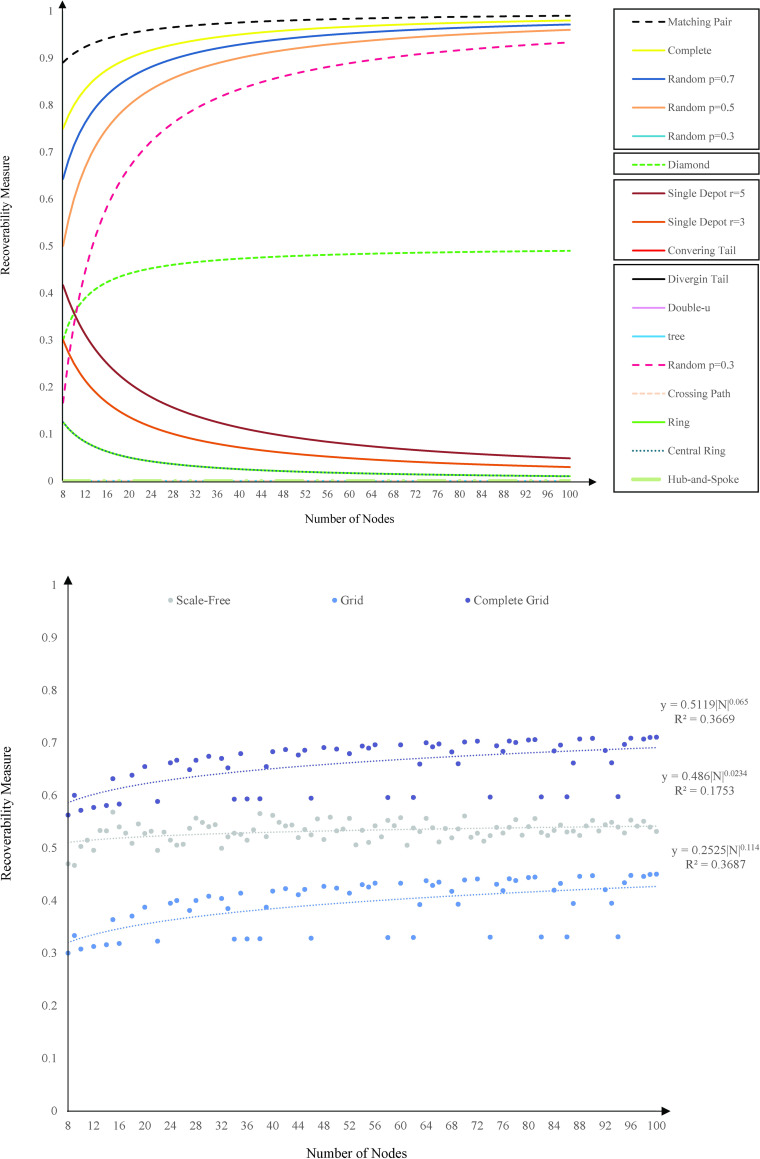
The analytical function of recoverability measure trends over size. (A) Diverging Tail, Hub-and-Spoke, Tree, Crossing Path, Double-U, Central Ring, Ring, Converging Tail, and Single Depot with 3 and 5 routes; Diamond, Matching Pair, Complete, and Random networks with connection probability of 0.3, 0.5, and 0.7. (B) Scale-Free, Grid, and Complete Grid networks.

Except for tree, diverging tails, crossing paths, and hub-and-spoke topologies that the recoverability measure remains the same by an increase in the size, our recoverability measure changes for other network topologies. Fig 6 depicts the analytical trend function of change in the recoverability measure by increasing the size of a network from 8 nodes to 100 nodes. As the size of a network is raised, different network topologies display different trends of our recoverability measure. In spite of the difference, the trend in their recoverability measure follows a particular mapping function $f_{}:(N,T)\to R$, where *f* is a representative function of nodes and topology, (*N*,*T*), and R is our recoverability measure. Here, we elaborate on the behavioral change of the recoverability measure in response to the size of the network for our 16 network topologies tested in this research. Our results indicate that diverging tail, hub-and-spoke, tree, and crossing path topologies require the recoverability of all links to return to their original performance as their directed topology includes no cycle. The recoverability measure then follows $f_{}(N)={1-\frac{|N|-1}{|N|-1}\approx 0}_{}$ function. The directed topology of double-u, central ring, ring, and converging tail includes at least a subset of nodes connected only through a directed cycle. For such cycles with *m* nodes, the recovery of *m*−1 links is sufficient to connect all nodes in the cycle. For these topologies, the recoverability measure function is $f_{}(N,r)={1-\frac{|N|-1}{\left|N\right|}}_{}$. The recoverability measure function for single depot topology follows $f_{}(N,r)={1-\frac{|N|-1}{|N|+r-1}}_{}$ where *r* is the number of cycles. The recoverability measure function for diamond network is $f_{}(N)={1-\frac{|N|-1}{2|N|-6}}_{}$, for matching pair is $f_{}(N)=1-\frac{|N|-1}{{\left|N\right|}^{2}}$, and for complete network is $f_{}(N)=1-\frac{|N|-1}{{\left|N\right|}^{2}}$. In a random network with |*N*| nodes, the probability of connecting each pair equals *p*. The average number of links in a random network equals $p\frac{\left|N|(|N|-1\right)}{2}$, and the recoverability measure function is $f_{}(N,p)=1-\frac{|N|-1}{p\frac{\left|N|(|N|-1\right)}{2}}\approx 1-\frac{2}{p|N|}$.

For diamond, random, complete, and matching pairs topologies, the recoverability measure function takes the downward concave trajectory. This means with an increase in the number of nodes, the recoverability measure increases at a decreasing rate. We interpret the increase in the recoverability measure as a result of the increase in the density of the network, and the decreasing rate as a result of increase in the Hamiltonian path of the network. For double-u, central ring, ring, and converging tail topologies, the recoverability measure function takes the upward concave trajectory. This means with an increase in the number of nodes, the recoverability measure decreases at a decreasing rate. Here, the increase in the network density is not as significant as the increase in the network Hamiltonian path and consequently results in a decrease in recoverability measure. The decreasing rate reflects the effect of increase in the network density, however insignificant, as the number of nodes increases. In the presence of thermodynamic limit, |*N*|→∞, the recoverability measure leans towards 0 for double-u, central ring, ring, converging tail, and single-depot, $\frac{1}{2}$ for diamond network, and 1 for matching pairs, complete, and random networks. This means the double-u, central ring, ring, converging tail, and single-depot networks become extremely vulnerable at large scale, and matching pairs, complete, and random networks become the most robust networks at large scale. The diamond network shows a constant recoverability measure starting from 36 nodes showing the independence of network recoverability from the size of the network.

Grid, complete grid, and scale-free topologies follow a different physical pattern of recoverability measure in response to the size of network, and as a result we plot their behavior separately in Fig 6. For the grid topology, the recoverability measure function follows $f_{}(N,n,m)=1-\frac{|N|-1}{n(m-1)+m(n-1)}$, and for complete grid topology the function is $f_{}(N,n,m)=1-\frac{|N|-1}{n(m-1)+m(n-1)+2(n-1)(m-1)}$, where *n*×*m* = |*N*|. For scale-free topology, there is no specific analytical function to identify the trends in recoverability measure. This happens due to the variations in the probability for adding a new node connected to an existing node in the scale-free network generation process.

In grid and complete grid topologies with size |*N*|, the topology of the network depends on the layout of nodes in rows, *n*, and columns, *m*, *n*×*m* = |*N*|. The layout affects the recoverability measure and consequently disturbs the consistency of trends in the recoverability measure. Despite the fluctuations, the recoverability measure of the grid, complete grid, and scale-free networks lean toward 1 at large scale, showing the robustness of the network against disruptions.

A grid network with |*N*| = 24, for instance, can be arranged as 4×6, 3×8, and 2×6 layout with recoverability measures 0.31, 0.70, and 0.71, respectively. This change in the layout of grid and complete grid network justifies the fluctuations in their corresponding trends. The trend line for grid and complete grid topologies follows a power function *f*(|*N*|) = 0.5119|*N*|^0.065^ for |*N*|≤1.43×10^11^ with *R*^2^ = 0.36 and *f*(|*N*|) = 0.2525|*N*|^0.114^ for |*N*|≤1.13×10^9^ with *R*^2^ = 0.36, respectively. Similar but not identical, we observe no trend in the scale-free network recoverability measure. Here, the best fitted trend line for the recoverability measure function is *f*(|*N*|) = 0.486|*N*|^0.0234^ for |*N*|≤8.94×10^31^ with *R*^2^ = 0.17.

### Recoverability and topology measures

We examine how the recoverability measure of a network is identified by the topology of the network and its measures. An individual network measure may characterize one or several aspects of the topology. We consider basic and important measures including betweenness centrality, average Shimbel, diameter, beta, and gamma. The measures detect aspects of connectivity and accessibility of networks.

We develop a set of bivariate linear regression models, where the recoverability measure is the dependent variable and the independent variables are the linear and square form of each network measure as expressed in Eq 13.

$$Y_{i}=\beta_{0}+\beta_{1}X_{i}+\beta_{2}{X_{i}}^{2}+u_{i}$$ (13)

Here, *Y*_*i*_ is the vector of endogenous variable, *X*_*i*_ is the vector of exogenous variables, *β*_0_, *β*_1_, and *β*_2_ are regression coefficients estimated by maximum likelihood, and *u*_*i*_ is the Gaussian distributed error term. The slope coefficient measures the marginal effects, which is the change in *Y* for a unit change in *X*. We judged the statistical significance of variables using P-value. We measured the goodness-of-fit of the models calculating the Adjusted *R*^2^, which ranges between 0 and 1 and the greater the value is the better the fit of model becomes. This index measures the improvement of a fitted model over a null model.

Table 3 reports the results of the modeling. Results indicate that our recoverability measure is significantly explained by betweenness centrality, beta, and gamma. Their Adjusted *R*^2^ fluctuates between 0.98 and 0.83. It means betweenness centrality, beta, and gamma explain between a 98% and 83% variation in the recoverability measure around its mean, having a similar explanation power for beta and gamma. The percentage of the recoverability measure variations that our models explain, however, diminishes with an increase in the size of network. While average Shimbel explains a 75% variation in the recoverability measure around its mean in networks with 8 nodes, the goodness-of-fit declines to 0.59 for networks with 26 nodes. The Adjusted *R*^2^ of the models including diameter is almost 0.55 regardless of the size of network. Unlike betweenness centrality, beta, gamma, and average Shimbel, the explanatory power of diameter measure remains partially the same with an increase in the size of network, indicating the robustness of the explanation.

**Table 3:** Results of the linear regression models with simple or quadratic form.

	Number of Nodes									
Coefficients	8	10	12	14	16	18	20	22	24	26
**Betweenness Centrality**										
***β***_**0**_	0.907	0.901	0.900	0.884	0.884	0.871	0.878	0.866	0.856	0.853
***β***_**1**_	-0.150	-0.110	-0.079	-0.062	-0.048	-0.039	-0.033	-0.027	-0.022	-0.020
***β***_**2**_	0.006	0.003	0.001	0.001	0.0006	0.0004	0.0003	0.0002	0.0001	0.0001
**Adj. *R***^**2**^	0.957	0.940	0.930	0.911	0.901	0.888	0.879	0.867	0.844	0.835
**Average Shimbel**										
***β***_**0**_	1.108	0.976	0.953	0.886	0.870	0.857	0.830	0.823	0.806	0.779
***β***_**1**_	-0.424	-0.302	-0.252	-0.203	-0.178	-0.156	-0.134	-0.121	-0.111	-0.099
**Adj. *R***^**2**^	0.758	0.707	0.694	0.661	0.638	0.646	0.627	0.633	0.617	0.592
**Diameter**										
***β***_**0**_	0.795	0.741	0.752	0.716	0.711	0.718	0.703	0.711	0.699	0.683
***β***_**1**_	-0.165	-0.120	-0.100	-0.082	-0.071	-0.064	-0.055	-0.050	-0.046	-0.041
**Adj. *R***^**2**^	0.549	0.552	0.562	0.550	0.516	0.547	0.529	0.544	0.529	0.514
**Beta**										
***β***_**0**_	-0.647	-0.533	-0.462	-0.427	-0.358	-0.314	-0.270	-0.233	-0.187	-0.173
***β***_**1**_	0.451	0.361	0.307	0.280	0.238	0.212	0.188	0.168	0.147	0.139
***β***_**2**_	-0.036	-0.023	-0.017	-0.0140	-0.010	-0.008	-0.006	-0.005	-0.004	-0.003
**Adj. *R***^**2**^	0.984	0.976	0.971	0.964	0.950	0.938	0.924	0.909	0.886	0.879
**Gamma**										
***β***_**0**_	-0.648	-0.534	-0.462	-0.427	-0.358	-0.314	-0.270	-0.233	-0.187	-0.173
***β***_**1**_	1.244	1.012	0.871	0.800	0.684	0.613	0.545	0.489	0.430	0.407
***β***_**2**_	-0.275	-0.187	-0.139	-0.114	-0.086	-0.070	-0.056	-0.046	-0.037	-0.033
**Adj. *R***^**2**^	0.984	0.977	0.971	0.964	0.950	0.939	0.924	0.909	0.886	0.879

Note: All variables are significant at the 99% confidence interval.

**Fig 7:**
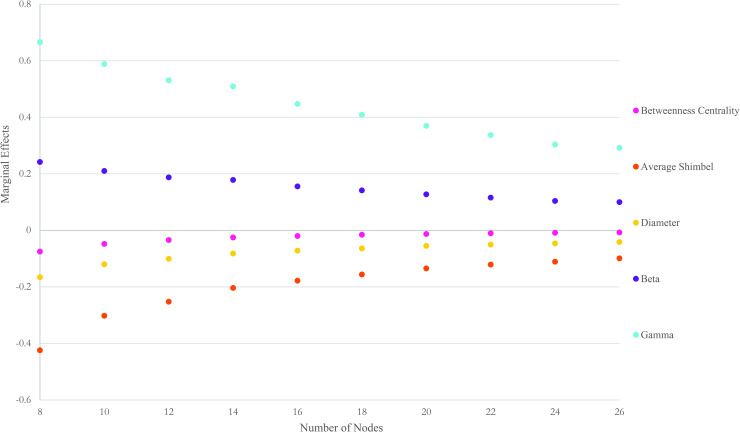
Marginal effects of topology measures on recoverability measure.

Fig 7 graphs the marginal effects of topology measures over the size of the network. Marginal effects measure the impact that an instantaneous unit change in an exogenous variable has on the endogenous variable. As shown, the highest positive effect is for the gamma measure. A one-unit increase in the ratio of the number of links to the maximum possible number of links increases the recoverability of the network, on average, by 0.44 over different sizes. The magnitude of a positive impact is tightly followed by beta measure. The marginal effects of this measure fluctuates between 0.24 and 0.09 with an average of 0.15 over different sizes. The highest negative correlation is for the average Shimbel index. A one-unit increase in the average of the sum of the lengths of all shortest paths connecting all pairs of nodes decreases the recoverability measure, on average, by 0.19 over different sizes. The magnitude of negative impact is followed by diameter measure. The marginal effects of this measure fluctuates between -0.16 and -0.04 with an average of -0.08 over different sizes. Two conclusions can be drawn from the marginal effects chart. First, regardless of the topology measure, the marginal effects diminish with an increase in the size of network. While the statistical correlation between recoverability and network measures is robust, the magnitude of impact is reduced when the size of a network increases. Second, the accessibility aspects of networks have a negative effect on the recoverability measure, unlike the connectivity aspects. By investigating the impact of betweenness centrality on recoverability measures over different sized networks, it is noted an increase in the number of times a node is crossed by the shortest path in the graph reduces the recoverability measure of the network.

## Conclusions

We argue that our analysis embodies the transition of natural and engineered systems from the disrupted state to the recovered state, and quantifies the effect of network topology on facilitating or impeding this transition. We have scrutinized the recovery behavior of networks with common topologies and different sizes, rather than self-selecting, both numerically and analytically with excellent agreement. We have developed satisfactory sizes under which recovery happens either quickly or slowly. The behavior is tight for multiple network topologies in small sized networks, while it diverges by an increase in the size of the network. This is justified by the increase in the average length of the shortest path in the diverging trajectories. We have shown that the disrupted network attains its recovered state gradually with an increasing return to recovery. This is justified by the uniform demand distribution between nodes. We speculate that the recovery might take a constant or decreasing shape of return as well, if the uniform demand distribution assumption is uplifted. This constitutes a promising future research avenue.

By scrutinizing the recovery behavior of networks, we have expanded the knowledge of recovery in networks with introducing and testing the recoverability measure. This measure is a function of network topology and is explained by the physics of the network. Our results have revealed that the network topology with high network connectivity follows a downward concave recoverability measure trend. Regardless of the network size, the recoverability measure trends for the network topologies with a low level of connectivity and no cycle equals zero. For networks with a low level of connectivity, the existence of at least one subset of nodes connecting only through a cycle turns the recoverability measure trends to downward concave functions. In between, there are some network topologies that do not follow any significant trend, including scale-free, grid, and complete grid topologies. The power trend line is the best illustrator of these networks recoverability measure behavior in response to an increase in the size of the network. Our recoverability measure developed here provides an understanding of how the recoverability of the network is a function of not only the size of the network, but also the connectivity and accessibility measures of the network. Our results show that a network becomes more recoverable as connectivity measures of the network such as beta and gamma increase. In particular, a network becomes more recoverable with an increase in the ratio of the number of links to the number of nodes and the ratio of the number of links to the maximum possible number of links. A network, however, becomes less recoverable as accessibility measures of the network, such as betweenness centrality, Shimbel index, and diameter, increase. De facto, accessibility decreases the recoverability of the system, as reflected in the lower recoverability measure and abrupt transitions. The functionality is not robust over the size of the network and the connection between the recoverability measure and topology measures declines with an increase in the size of the network. Although our measure is applied here to study the recoverability of unweighted networks, it is expandable to the study of weighted networks with an uneven demand distribution.

Overall, the results not only offer guidance on designing recoverable networks, but also picture the recovery nature of networks deliberately following a disruption. The knowledge of network recovery helps assess the system risks and govern system mitigation under random attacks, malicious attacks, and the localized attacks induced by natural disasters or mass attacks. Our recovery behavior and recoverability measure has been tested on 16 distinct network topologies. The relevant recovery behavior can be generalized based on our definition for any network topology recovering deliberately.

## Software and packages

The network topologies are coded in Python 3.7.3, and we use Gurobi 9.0.1 package to solve the recoverability model. All instances were tested on an Intel® Core i7-8700U CPU @ 3.20 GHz 3.19 GHz with 64 GB RAM. The codes for generating network topologies and characteristics are publicly available via https://github.com/MODALHUB/NetworkResilence for use and distribution, except for commercial use, with proper citations and acknowledgement of the authors.

## Supporting information

S1 File NP-hardness of problems with *O*(*n*!) complexity. (DOCX)

S1 Fig Recoverability trajectory of 16 network topologies. (A) 8 nodes, (B) 10 nodes, (C) 12 nodes, (D) 14 nodes, (E) 16 nodes, (F) 18 nodes, (G) 20 nodes, (H) 22 nodes, (I) 24 nodes, (J) 26 nodes.
(ZIP)
