# Supplementary material for: Recovery patterns and physics of the network
Source: PLoS One. 2021 Jan 19;16(1):e0245396. doi: 10.1371/journal.pone.0245396 (PMC7815135; doi:10.1371/journal.pone.0245396)
Supplement: S1 File — (DOCX) [file pone.0245396.s001.docx]

**S1 NP-Hardness of problems with** $\boldsymbol{O(n!)}$ **complexity**

We define $O(.)$ as a mathematical notation to describe how fast the complexity of a model increases when the number of possible decisions tends towards a particular value or infinity. The complexity of our proposed method equals $O(n!)$, where we sort the disrupted links in terms of receiving restoration service one at a time.

For models with a high level of $O(.)$, we cannot prove that a polynomial solution time exists. In other words, it takes a computationally infinite time to solve a model when its possible decisions tend toward infinity. Such a class of models is called NP-Hard. As an example, the zero-one knapsack problems are known to be an NP-Hard problem.

In a zero-one knapsack problem, we are given $n$ number of items, each of which has a specific weight $w_{i}$, where $i=1,\ldots,n$. We can pick $m$ number of items $(m\leq n)$ out of the given $n$ items. The goal is to choose the $m$ number of items in such a way that the aggregate weight is maximized while it does not exceed a specific value ($W$). The complexity of zero-one knapsack problems equals $O(2^{n})$, where each of the $n$ items can be chosen or otherwise. Using a mathematical induction, we prove that the problems with $O(n!)$ notation are more complicated than problems with $O(2^{n})$ notation, and therefore they are NP-Hard. We prove adding one unit to a specific value of $n$ increases the solution time of a problem with $O(n!)$ complexity considerably more than the solution time of a zero-one knapsack problem with $O(2^{n})$ complexity. The following is the mathematical representation of the induction proof method:

| $Step 1: n>2 \forall n>2$  $\vdots$  $Step n: n!>2^{n} \forall n>2$  $Step n+1: \left( n+1 \right)\times n!>\left( n+1 \right)\times2^{n}>2\times2^{n}\to\left( n+1 \right)!>2^{n+1} \forall n>2$ |  |
| --- | --- |

As shown, $O\left( n! \right)>O\left( 2^{n} \right)$. It means the computational complexity of our proposed model is higher than a zero-one knapsack problem, and consequently, it is an NP-hard problem.
