# Supplementary figures and images for: Recovery patterns and physics of the network
Source: PLoS One. 2021 Jan 19;16(1):e0245396. doi: 10.1371/journal.pone.0245396 (PMC7815135; doi:10.1371/journal.pone.0245396)

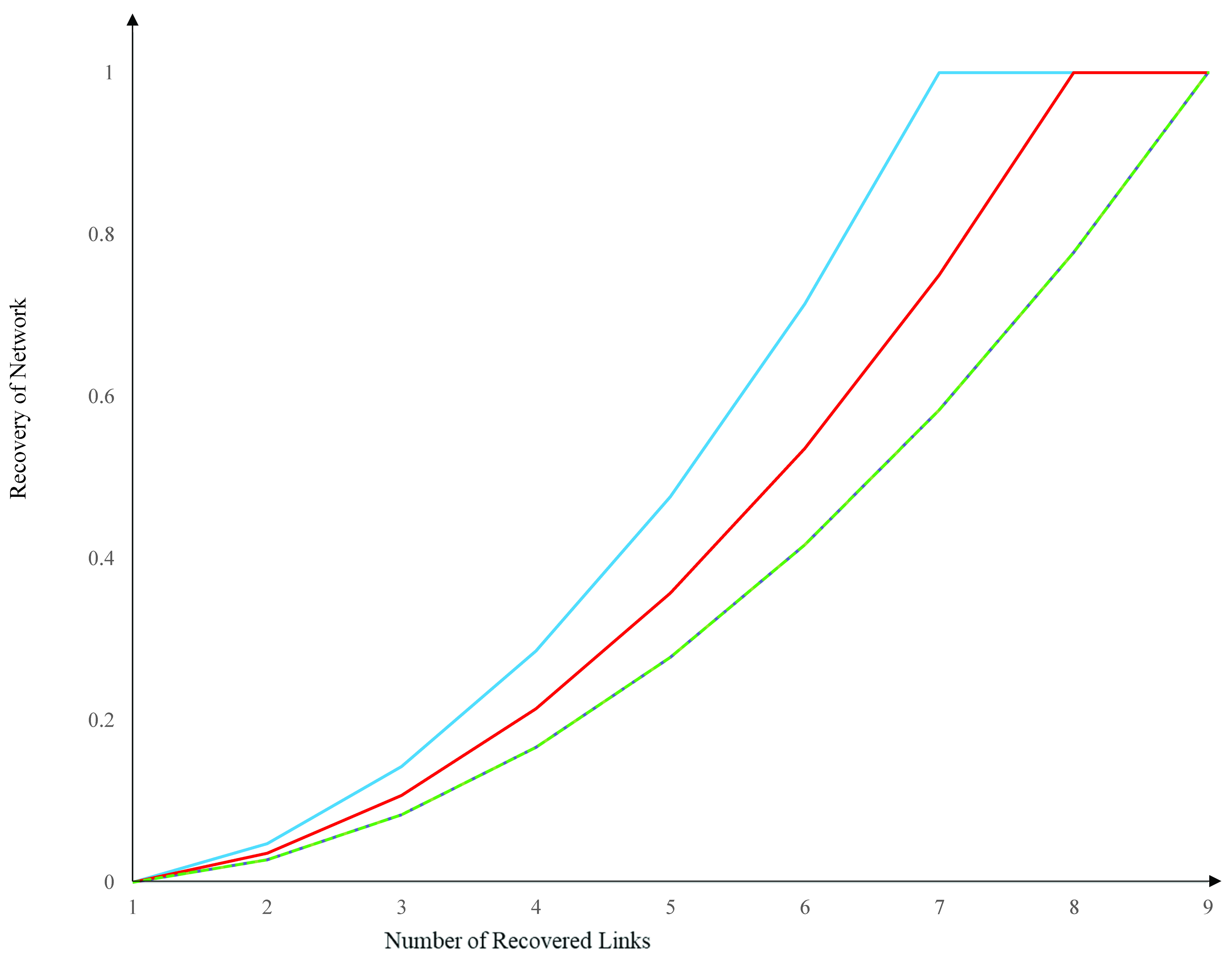

Supplement: S1 Fig — (A) 8 nodes, (B) 10 nodes, (C) 12 nodes, (D) 14 nodes, (E) 16 nodes, (F) 18 nodes, (G) 20 nodes, (H) 22 nodes, (I) 24 nodes, (J) 26 nodes. (ZIP) [file pone.0245396.s002.zip › S1a_Fig.tif]

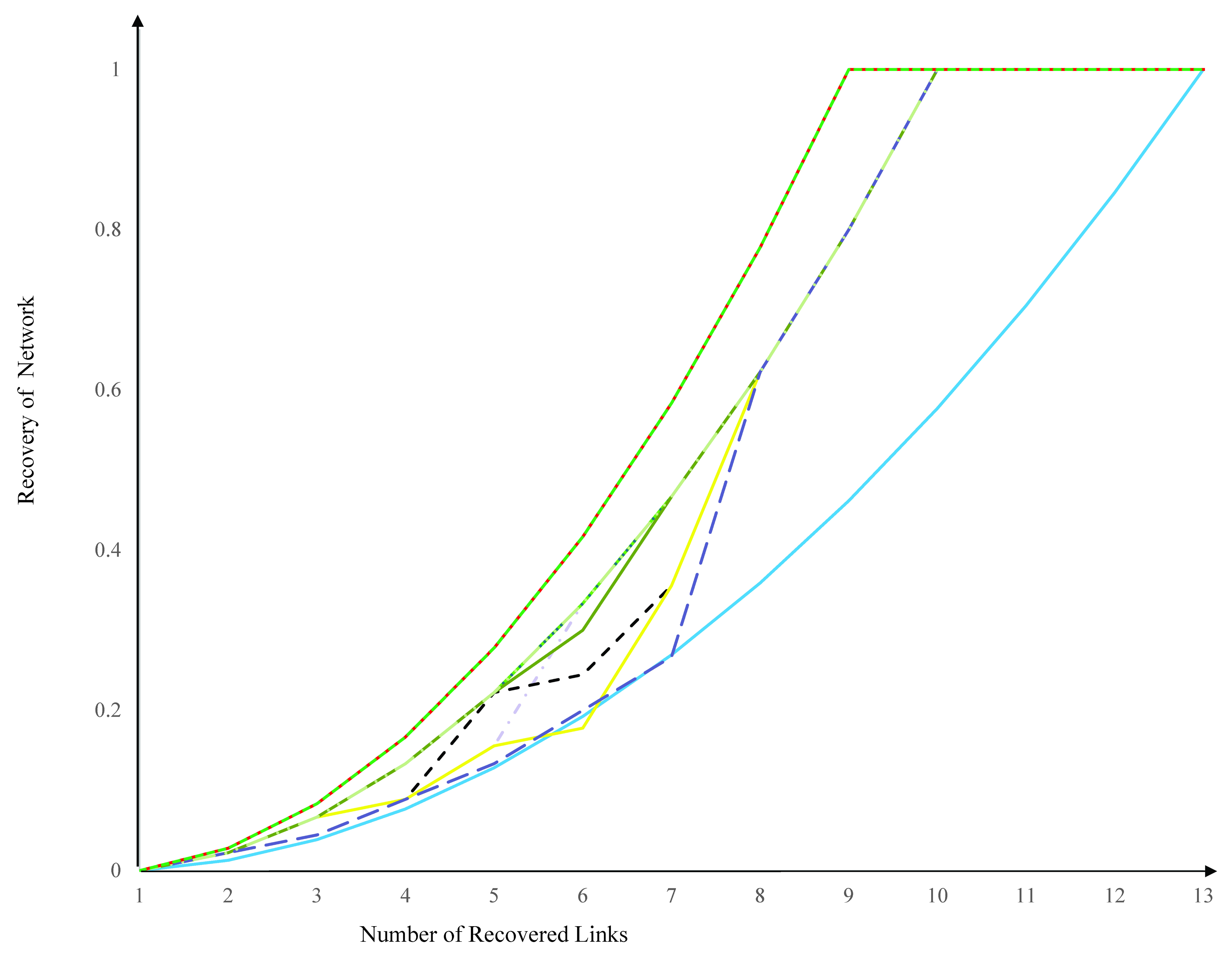

Supplement: S1 Fig — (A) 8 nodes, (B) 10 nodes, (C) 12 nodes, (D) 14 nodes, (E) 16 nodes, (F) 18 nodes, (G) 20 nodes, (H) 22 nodes, (I) 24 nodes, (J) 26 nodes. (ZIP) [file pone.0245396.s002.zip › S1b_Fig.tif]

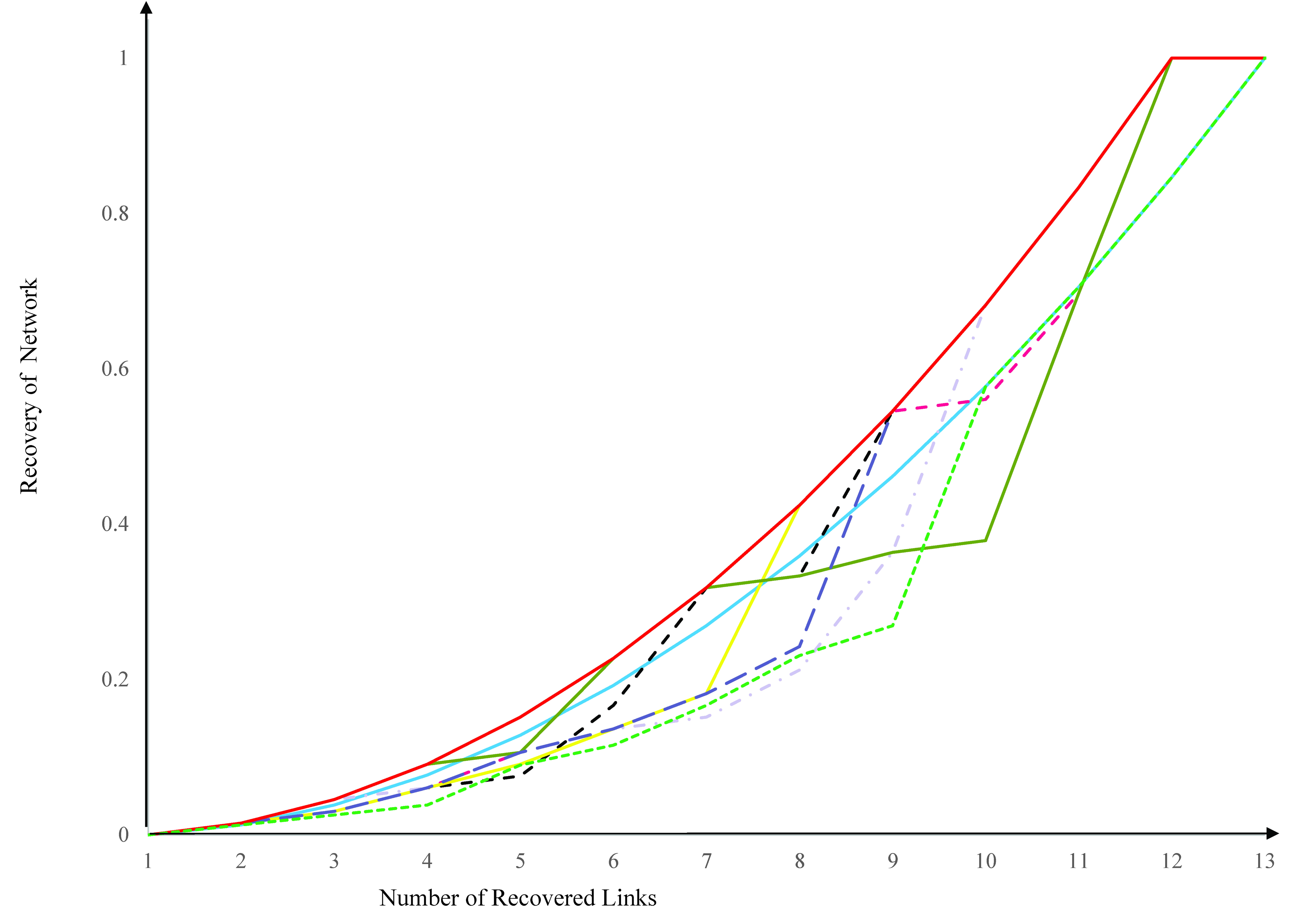

Supplement: S1 Fig — (A) 8 nodes, (B) 10 nodes, (C) 12 nodes, (D) 14 nodes, (E) 16 nodes, (F) 18 nodes, (G) 20 nodes, (H) 22 nodes, (I) 24 nodes, (J) 26 nodes. (ZIP) [file pone.0245396.s002.zip › S1c_Fig.tif]

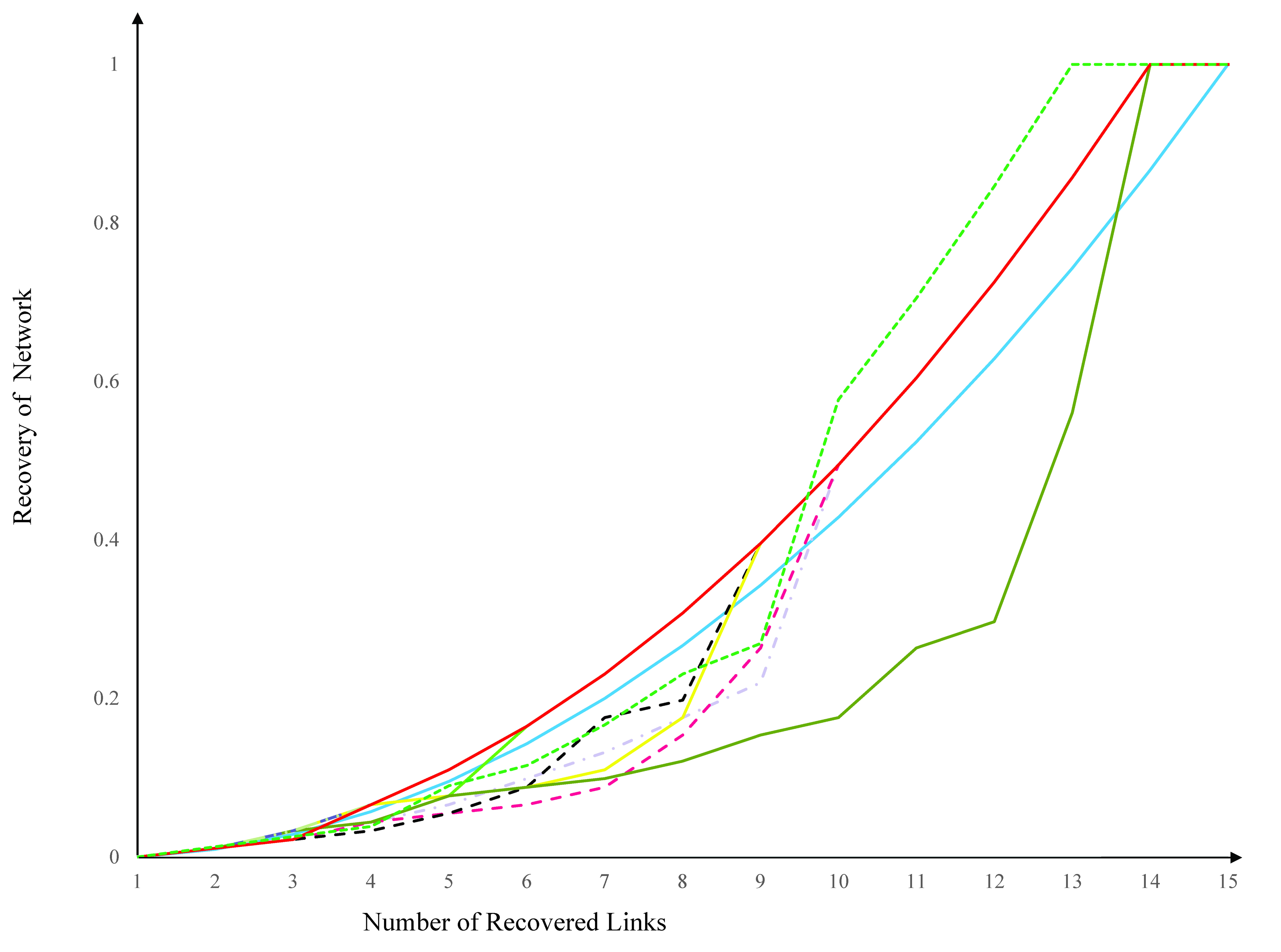

Supplement: S1 Fig — (A) 8 nodes, (B) 10 nodes, (C) 12 nodes, (D) 14 nodes, (E) 16 nodes, (F) 18 nodes, (G) 20 nodes, (H) 22 nodes, (I) 24 nodes, (J) 26 nodes. (ZIP) [file pone.0245396.s002.zip › S1d_Fig.tif]

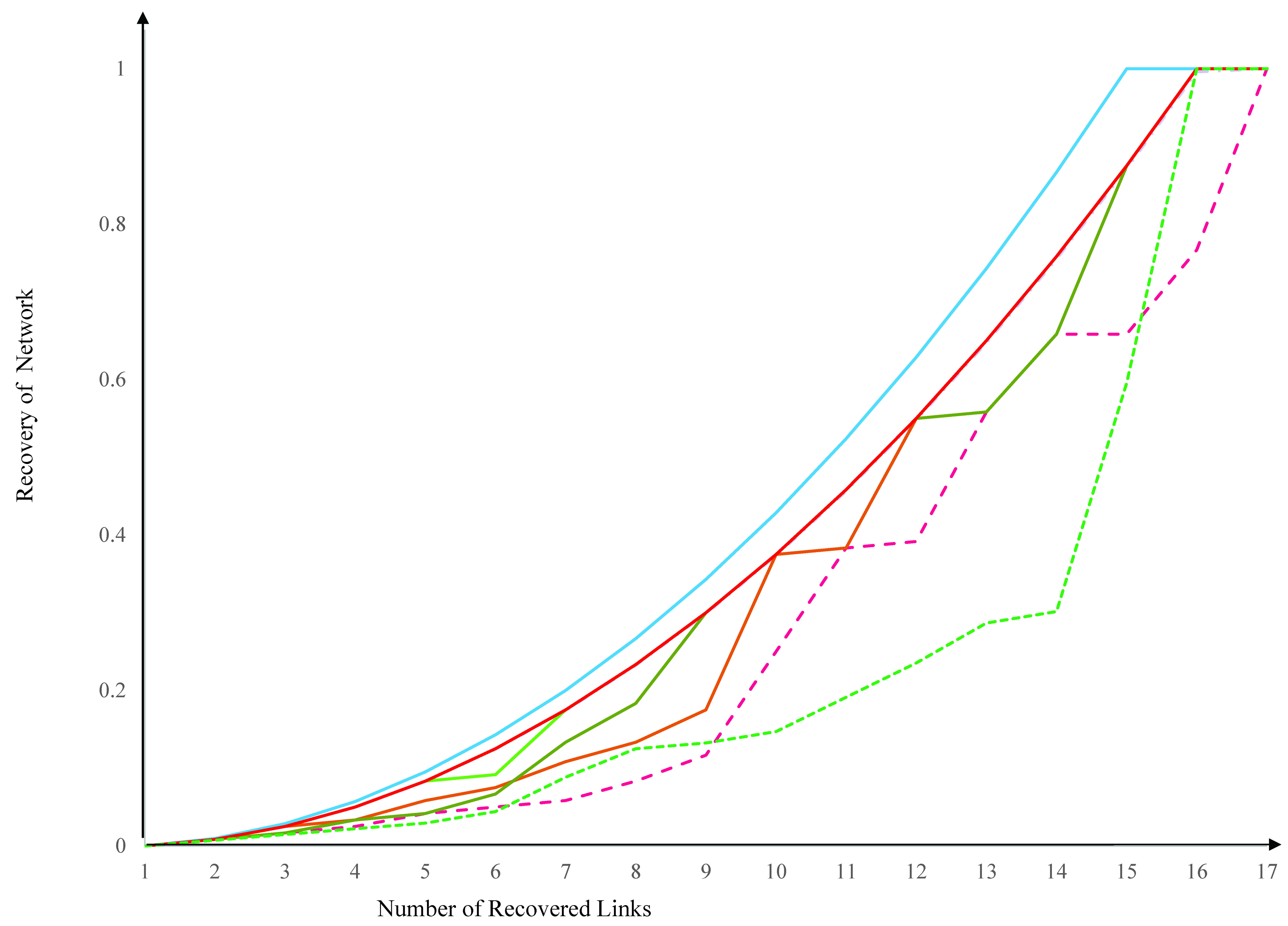

Supplement: S1 Fig — (A) 8 nodes, (B) 10 nodes, (C) 12 nodes, (D) 14 nodes, (E) 16 nodes, (F) 18 nodes, (G) 20 nodes, (H) 22 nodes, (I) 24 nodes, (J) 26 nodes. (ZIP) [file pone.0245396.s002.zip › S1e_Fig.tif]

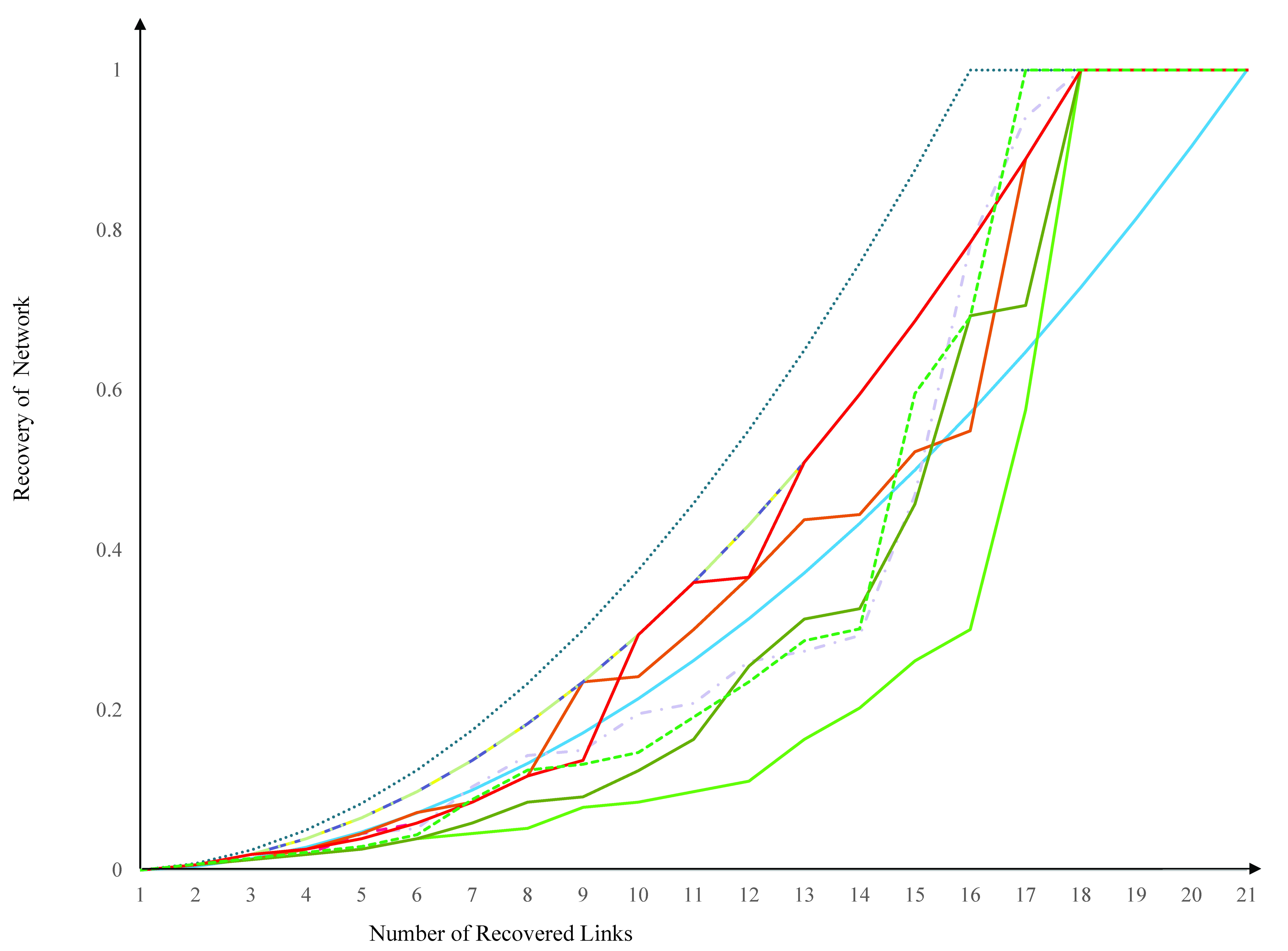

Supplement: S1 Fig — (A) 8 nodes, (B) 10 nodes, (C) 12 nodes, (D) 14 nodes, (E) 16 nodes, (F) 18 nodes, (G) 20 nodes, (H) 22 nodes, (I) 24 nodes, (J) 26 nodes. (ZIP) [file pone.0245396.s002.zip › S1f_Fig.tif]

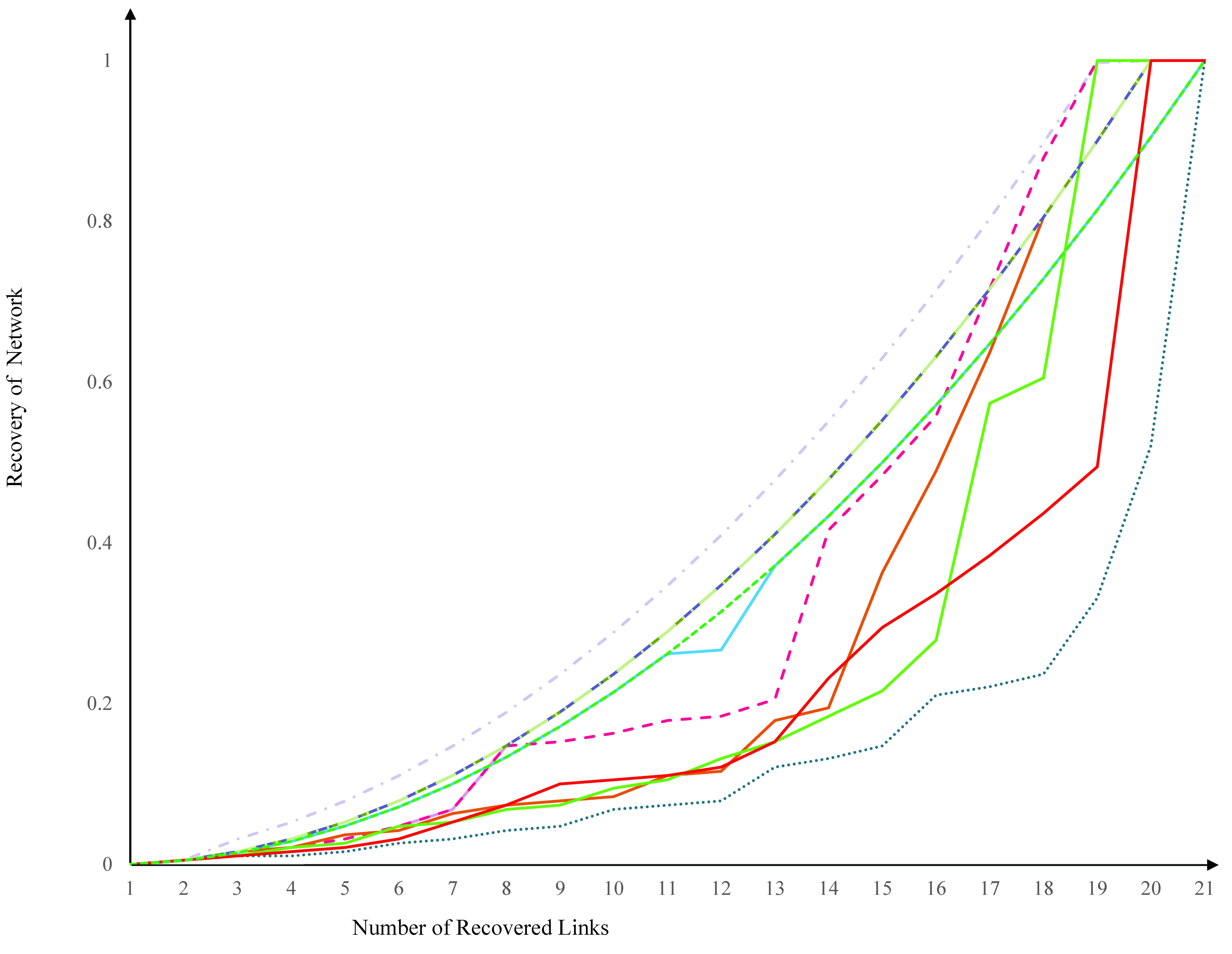

Supplement: S1 Fig — (A) 8 nodes, (B) 10 nodes, (C) 12 nodes, (D) 14 nodes, (E) 16 nodes, (F) 18 nodes, (G) 20 nodes, (H) 22 nodes, (I) 24 nodes, (J) 26 nodes. (ZIP) [file pone.0245396.s002.zip › S1g_Fig.tif]

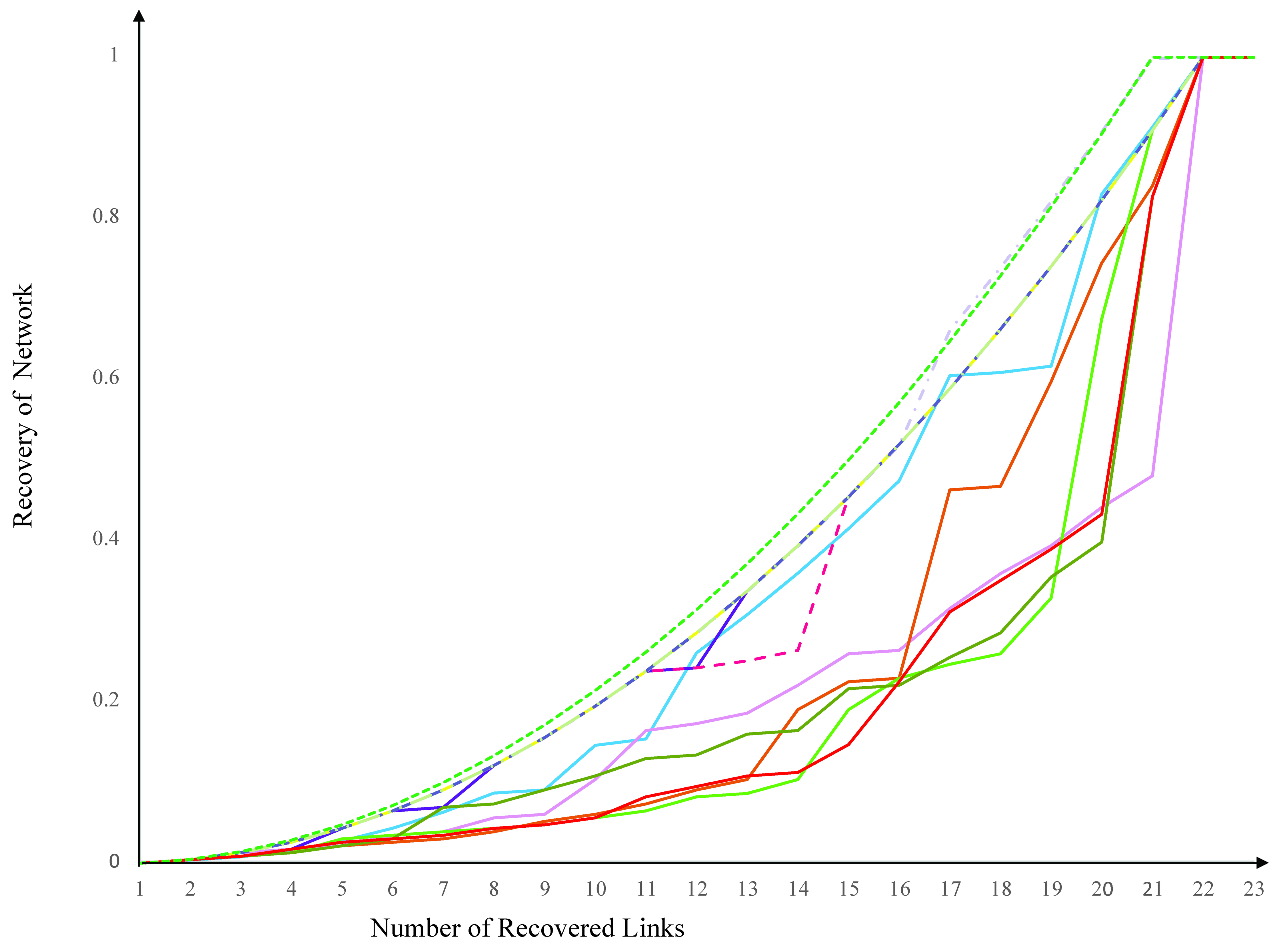

Supplement: S1 Fig — (A) 8 nodes, (B) 10 nodes, (C) 12 nodes, (D) 14 nodes, (E) 16 nodes, (F) 18 nodes, (G) 20 nodes, (H) 22 nodes, (I) 24 nodes, (J) 26 nodes. (ZIP) [file pone.0245396.s002.zip › S1h_Fig.tif]

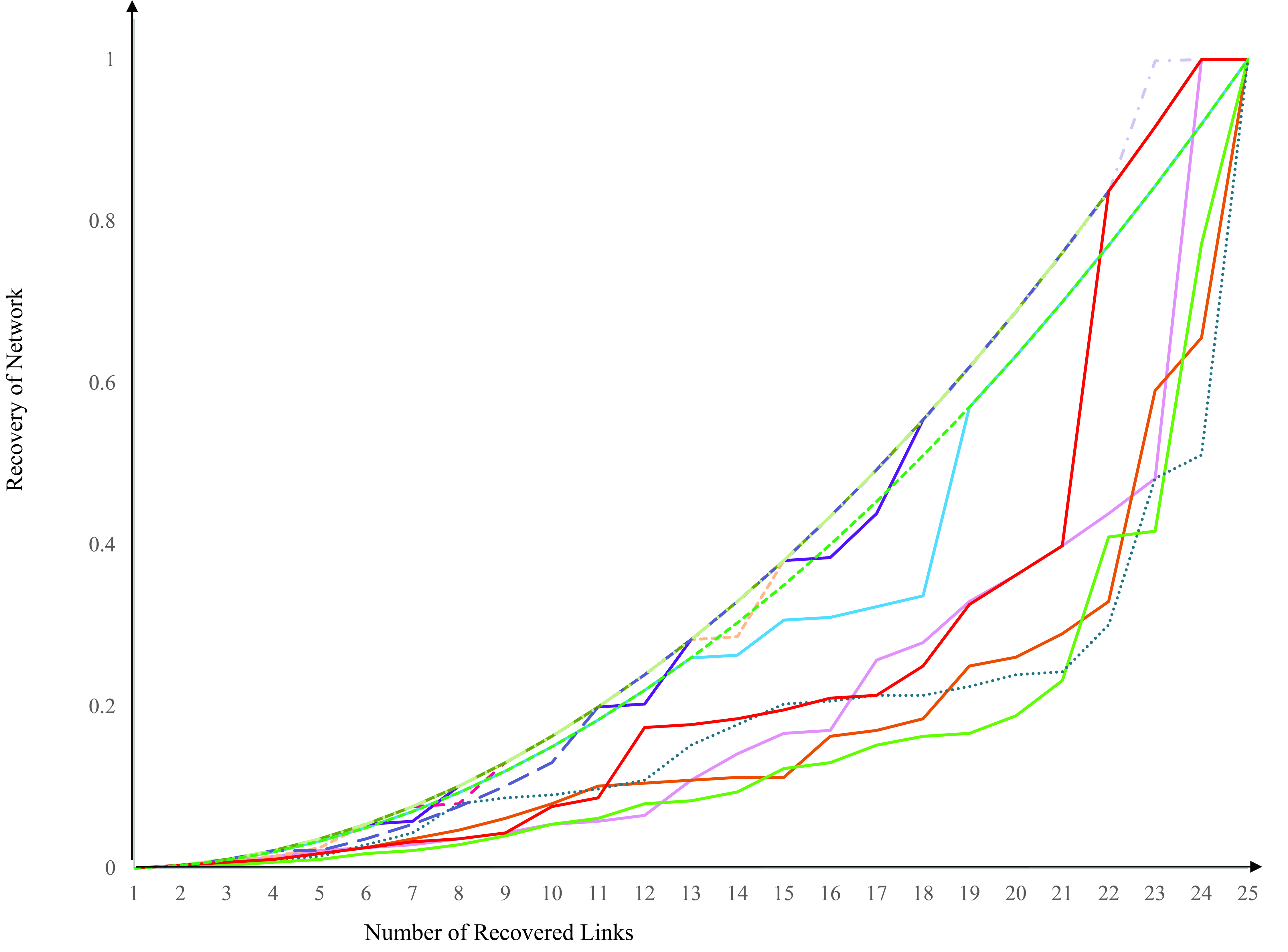

Supplement: S1 Fig — (A) 8 nodes, (B) 10 nodes, (C) 12 nodes, (D) 14 nodes, (E) 16 nodes, (F) 18 nodes, (G) 20 nodes, (H) 22 nodes, (I) 24 nodes, (J) 26 nodes. (ZIP) [file pone.0245396.s002.zip › S1i_Fig.tif]

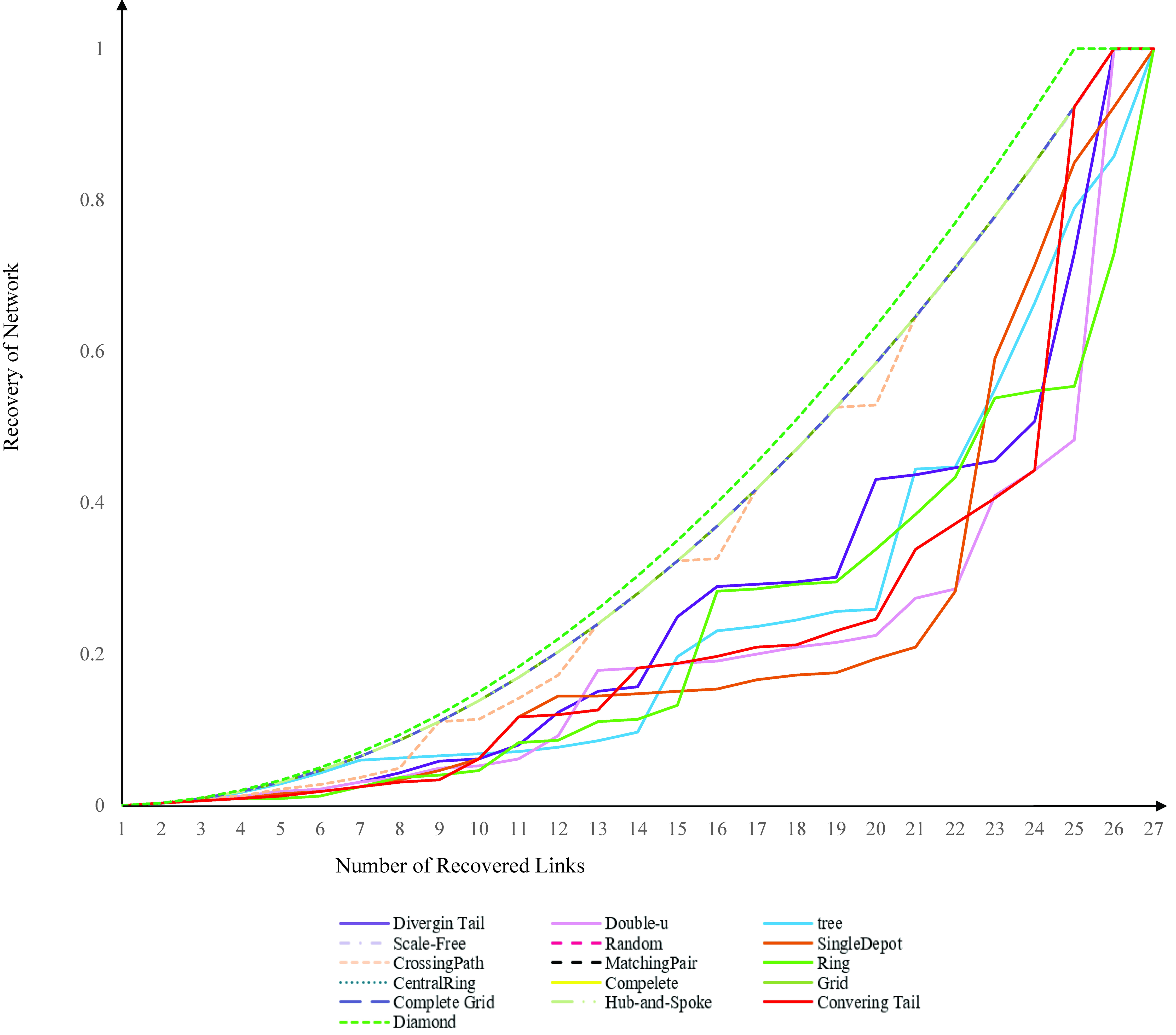

Supplement: S1 Fig — (A) 8 nodes, (B) 10 nodes, (C) 12 nodes, (D) 14 nodes, (E) 16 nodes, (F) 18 nodes, (G) 20 nodes, (H) 22 nodes, (I) 24 nodes, (J) 26 nodes. (ZIP) [file pone.0245396.s002.zip › S1j_Fig.tif]
